# BRCA1-Associated RING Domain-1 (BARD1) Loss and GBP1 Expression Enhance Sensitivity to DNA Damage in Ewing Sarcoma

**DOI:** 10.1158/2767-9764.CRC-21-0047

**Published:** 2022-04-20

**Authors:** Lisa M. Maurer, Jessica D. Daley, Elina Mukherjee, Rosemarie E. Venier, Claire M. Julian, Nathanael G. Bailey, Michelle F. Jacobs, Chandan Kumar-Sinha, Haley Raphael, Nivitha Periyapatna, Kurt Weiss, Katherine A. Janeway, Rajen Mody, Peter C. Lucas, Linda M. McAllister-Lucas, Kelly M. Bailey

**Affiliations:** 1 Department of Pediatrics, University of Pittsburgh School of Medicine, Pittsburgh, Pennsylvania.; 2 Department of Human Genetics, Graduate School of Public Health, University of Pittsburgh, Pittsburgh, Pennsylvania.; 3 Department of Pathology, University of Pittsburgh School of Medicine, Pittsburgh, Pennsylvania.; 4 Department of Internal Medicine, University of Michigan Medical School, Ann Arbor, Michigan.; 5 Michigan Center for Translational Pathology, Ann Arbor, Michigan.; 6 Department of Orthopedic Surgery, University of Pittsburgh School of Medicine, Pittsburgh, Pennsylvania.; 7 Pediatric Oncology, Dana-Farber / Boston Children's Cancer and Blood Disorders Center, Boston, Massachusetts.; 8 Department of Pediatrics, University of Michigan Medical School, Ann Arbor, Michigan.

## Abstract

Ewing sarcoma is a fusion oncoprotein–driven primary bone tumor. A subset of patients (∼10%) with Ewing sarcoma are known to harbor germline variants in a growing number of genes involved in DNA damage repair. We recently reported our discovery of a germline mutation in the DNA damage repair protein *BARD1* (BRCA1-associated RING domain-1) in a patient with Ewing sarcoma. BARD1 is recruited to the site of DNA double stranded breaks via the PARP protein and plays a critical role in DNA damage response pathways including homologous recombination. We thus questioned the impact of BARD1 loss on Ewing cell sensitivity to DNA damage and the Ewing sarcoma transcriptome. We demonstrate that PSaRC318 cells, a novel patient-derived cell line harboring a pathogenic *BARD1* variant, are sensitive to PARP inhibition and by testing the effect of BARD1 depletion in additional Ewing sarcoma cell lines, we confirm that BARD1 loss enhances cell sensitivity to PARP inhibition plus radiation. In addition, RNA-sequencing analysis revealed that loss of *BARD1* results in the upregulation of *GBP1* (guanylate-binding protein 1), a protein whose expression is associated with variable response to therapy depending on the adult carcinoma subtype examined. Here, we demonstrate that GBP1 contributes to the enhanced sensitivity of BARD1-deficient Ewing cells to DNA damage. Together, our findings demonstrate the impact of loss-of function mutations in DNA damage repair genes, such as *BARD1*, on Ewing sarcoma treatment response.

**Significance:**

This work provides preclinical support for the inclusion of pediatric patients with advanced Ewing sarcoma and pathogenic germline variants in *BARD1* in future clinical trials testing novel agents inducing DNA damage/targeting DNA damage repair.

## Introduction

Ewing sarcoma is a primary bone cancer driven by an aberrant fusion between *EWSR1* and a gene encoding an E26 transformation–specific (ETS) transcription factor, most commonly *FLI1* (1). Mechanistically, EWS-FLI1 fusions are formed as the result of either reciprocal translocation or chromoplexy events (2). Recent studies have revealed that EWS-FLI1 itself impairs homologous recombination (HR) by sequestering the HR protein BRCA1 (breast cancer gene 1; ref. 3). The disruption of HR by EWS-FLI1 supports the categorization of Ewing sarcoma as a “BRCAness” tumor, phenotypically mimicking loss of BRCA1 expression (4). Clinically, Ewing tumors demonstrate sensitivity to DNA-damaging agents such as doxorubicin (5). Preclinical studies using Ewing sarcoma cell lines and Ewing xenografts have demonstrated tumor sensitivity to compounds that prevent DNA damage repair such as PARP1 inhibitors (PARPi; refs. 6–8).

In addition to the DNA damage repair defects imparted by EWS-FLI1 itself, germline pathogenic variants in DNA damage repair genes, such as *APC*, *BRCA1*, *FANCC*, and *RAD51*, have been identified in greater than 10% of patients with Ewing sarcoma (9, 10). We recently contributed to this growing body of literature by reporting our discovery of a paternally inherited germline frameshift pathogenic variant in the RING domain of *BARD1* (BRCA1-associated RING domain protein 1) in a patient with Ewing sarcoma (11). BARD1, a known tumor suppressor, is an obligatory binding partner of BRCA1. The BRCA1-BARD1 heterodimer functions as an E3 ubiquitin ligase and promotes DNA double-strand break repair by HR (12). The contribution of germline pathogenic variants in DNA damage repair genes, such as *BARD1*, to the overall sensitivity of Ewing tumors to DNA damage is largely unknown. Patients with pathogenic BRCA1/2 variant hereditary breast and ovarian cancers demonstrate significant response to PARPi/DNA-damaging combinations, a response not seen in patients without these germline variants (13, 14). Thus, we questioned whether the subset of patients with Ewing sarcoma who also harbor germline pathogenic variants in DNA damage repair genes, such as a germline *BARD1* mutation, may demonstrate enhanced sensitivity to PARPi/DNA-damaging agent combinations.

Here, we demonstrate that additional hits to tumor DNA damage machinery, such as loss of BARD1 expression, can indeed render Ewing cells more sensitive to DNA-damaging agents, especially when in combination. Using an unbiased RNA-sequencing (RNA-seq) approach, we determined the impact of BARD1 loss on the Ewing cell transcriptome. We found that guanylate-binding protein 1 (GBP1) is significantly upregulated upon BARD1 downregulation and we subsequently demonstrate a role for GBP1 in DNA damage sensitivity in Ewing sarcoma.

## Materials and Methods

### Reagents

Antibodies were purchased from the following sources: anti-CD99 FITC conjugated (BD Biosciences, catalog no: 555688, concentration 1:20), anti-FLI1 (Abcam, catalog no: 133485, concentration 1:2,000), anti-BARD1 (Bethyl Laboratories, catalog no: A300–263A, 1:2,000), anti-BARD1 (Abcam, catalog no: ab50984, concentration 1:100), anti-GBP1 (Abcam, catalog no: 131255, concentration 1:300 for IHC), anti-GBP1 (Santa Cruz Biotechnology, catalog no: sc-53857, concentration 1:200 for Western blot analysis), anti-phospho (Ser 139)-γH2A.X (Millipore Sigma, catalog no: 05–636, concentration 1:2,500 for immunofluorescence), anti-phospho (Ser 139)-γH2A.X (Invitrogen, catalog no: MA1–2022, concentration 1:1,000 for Western blot analysis), goat anti-mouse IgG AF-488 (Thermo Fisher Scientific, catalog no: A-11001, concentration 1:2,000), tubulin (Cell Signaling Technology, catalog no: 2144S, concentration 1:5,000), vinculin (Cell Signaling Technology, clone E1E9V, catalog no: 13901S, concentration 1:5,000), and anti-rabbit IgG-horseradish peroxidase (HRP) (Promega, catalog no: W401B). Additional specialized reagents include: BMN 673 (talazoparib; Cayman Chemical, catalog no: 19782), MK-4827 tosylate (niraparib; Cayman Chemical, catalog no: 20842), DMSO (MP Biomedicals, catalog no: 196055), doxorubicin (Cayman Chemical, catalog no: 15007), and IFNγ (R&D systems, cat no: 285IF100).

### Patient-Derived Relapsed Ewing Sarcoma Cell Line

We have previously described an adolescent patient with relapsed Ewing sarcoma and a paternally inherited, pathogenic germline *BARD1* variant (11). This tumor was confirmed as Ewing sarcoma both by EWSR1 FISH and panel sequencing of the tumor. Sequencing revealed a type 3 EWSR1::FLI1 fusion (11). The PSaRC318 patient tumor–derived Ewing sarcoma cell line was generated by our laboratory (studies were approved by the University of Pittsburgh Institutional Review Board, STUDY19030108 and patient written informed consent for sample collection was obtained by the Musculoskeletal Oncology Biobank and Tumor Registry, STUDY20010034; studies were conducted in accordance with U.S. Common Rule ethical guidelines). Briefly, viably preserved tumor biopsy tissue was placed in warm Iscove's modified Dulbecco's medium (IMDM) supplemented with 20% heat-inactivated FBS. The tumor was then physically dissociated using a disposable stainless-steel blade, and individual tumor pieces were embedded in growth factor–reduced Matrigel (Corning). The dissociated tumor was incubated at 37^ο^C and 5% CO_2_, with media exchanged every 72 hours. Tumor organoids grew over the next 3 weeks and were then harvested. The PSaRC318 Ewing sarcoma cell line was generated by: generating single-cell suspensions, culturing cells on fibronectin-coated (R&D Systems, catalog no: 342000101) plates and serially depleting cultures of fibroblasts. Short tandem repeat (STR) profiling (University of Arizona Genetics Core, Tucson, AZ) was performed on the PSaRC318 cell line to confirm cell authentication prospectively.

### Additional Cell Lines and Culture Conditions

A673 and TC71 cells were cultured in RPMI + l-glutamine media supplemented with 10% FBS. CHLA9 and CHLA10 cells were cultured in IMDM + l-glutamine media supplemented with 20% FBS and 1% insulin–transferrin–selenium (ITS, R&D Systems, catalog no: AR013). Early passage stocks of cell lines were obtained in collaboration with the Lawlor laboratory (University of Michigan, Ann Arbor, MI, 2016) and were maintained at 37^ο^C and 5% CO_2_. All cell lines underwent routine STR profiling for cell authentication (University of Arizona Genetics Core, Tucson, AZ) and regular monitoring for *Mycoplasma* contamination (MycoAlert PLUS Mycoplasma Detection Kit, Lonza, catalog no:LT07–703).

### Radiation

Cells were radiated with X-RAD 320 (Precision X-ray Inc) using Filter 2 (1.5 mm Al + 0.25 mm Cu +0.75 mm Sn) at doses (Gy) noted in individual experiments.

### siRNA

BARD1 knockdown in Ewing sarcoma cells was achieved using *BARD1* siRNA SMART pool (Dharmacon, catalog no: L-003873–00–0005), which consists of four individual siRNAs targeting *BARD1* (catalog nos: J-003873–12–0005, J-003873–11–0005, J-003873–10–0005, and J-003873–09–0005). GBP1 knockdown was achieved by using *GBP1* siRNA SMART pool (Dharmacon, catalog no: L-005153–00–0005). ON-TARGET plus nontargeting pool (Dharmacon, catalog no: D-001810–10–2) was used as the control for all siRNA-based experiments. Briefly, 500 μL of optiMEM (Gibco, catalog no: 11058021) was placed per well (6-well plate) along with 3 μL of 20 μmol/L siRNA (reconstituted as per manufacturer's instructions using 5× siRNA buffer, Dharmacon, catalog no: B-002000-UB-100) and 2 μL (A673 and PSaRC318) or 2.5 μL (CHLA10) Liopfectamine RNAiMAX transfection reagent (Life Technologies, catalog no: 13778150). The mixture was incubated with intermittent rocking for 30 minutes prior to the addition of cells.

### RT-PCR

RNA isolation was performed using Qiagen RNeasy Plus Mini isolation kit (Qiagen, catalog no: 74134) for >500K cells or Qiagen RNeasy Plus Micro isolation kit (Qiagen, catalog no: 74034) for ≤500K cells. RNA concentration was measured using Nanodrop (Thermo Fisher Scientific). cDNA synthesis was performed on 1 μg of RNA with the High-Capacity cDNA Reverse Transcription Kit (Thermo Fisher Scientific, catalog no: 4374966) and Applied Biosystems Veriti 96-well Thermocycler. qRT-PCR analysis was performed using Taqman probes (Life Technologies, *GAPDH* Hs02758991_g1, *RPLP0* Hs00420895_gH, *BARD1* Hs00957655_m1, and *GBP1* Hs00977005_m1), Taqman Universal PCR Master Mix (Life Technologies, catalog no: 4304437), and StepOnePlus Real-Time PCR system (Life Technologies).

### Immunoblotting

Immunoblot analysis was performed on cell lysates prepared using RIPA or LDS lysis buffer as previously reported (15). After sonication, gel electrophoresis was performed using SDS-PAGE gel electrophoresis. After transfer to nitrocellulose, membranes were blocked with 5% milk in TBST and incubated with primary antibody overnight in 2.5%–5% milk (or BSA for phospho-antibodies)/TBST at 4°C. After washing, membranes were incubated with HRP-conjugated secondary antibody prior to the addition of ECL reagent (Thermo Scientific) and film exposure/processing. ImageJ software (https://imagej.nih.gov) was used to perform densitometry analysis of resulting bands.

### Flow Cytometry

Adherent Ewing sarcoma cells were detached from the culture plate using Accutase Cell Detachment Solution (Corning, catalog no: 25–058-Cl). Cells were first stained with Live/Dead Aqua (Life Technologies, #L34957) using a ratio of 1 μL stain per 1 × 10^6^ cells in 1 mL. Cells were then stained for CD99 (FITC, BD Biosciences, catalog no: 555688) using 5 μL of antibody per 1 × 10^6^ cells in 1 mL in a total volume of 100 μL. The percentage of live, FITC-positive cells and the mean fluorescence intensity was determined using a BD FACSAria III or BD FACSAria-II SORP. FlowJo software was used for data analysis and generation of data plots.

### Immunofluorescence Staining and Confocal Microscopy

Cells were fixed and immunofluorescently labeled as previously described (16). Slides were mounted using a DAPI-containing mounting solution and cells were imaged using Zeiss LSM 510 and Leica Stellaris 5 confocal microscopes.

### IHC

Formalin-fixed, paraffin-embedded (FFPE) Ewing tumor samples underwent deparaffinization, antigen retrieval (buffer pH 8.5–9, catalog no: 950–224), and staining for GBP1 (1:300 dilution, Abcam, catalog no: 131255) via IHC using a RUO DISCOVERY Multimer V2 (v0.00.0083) and Discovery ULTRA Staining Module (Department of Pathology, UPMC ISH Laboratory). Antibody dilution was selected in consultation with a pathologist following stained slide review.

### Live-Cell Monitoring and Apoptosis Assays

Cells were seeded into 96-well plates (Corning 3610 or 3596) in 100 μL of Fluorobrite media (Gibco, #A18967–01) containing 5% FBS minimally in triplicate. Because of baseline differences in proliferation between cell types, A673, CHLA10, and PSaRC 318 cells were seeded at a starting cell count of 5,000, 8,000, or 10,000 cells per well, respectively. Cell treatment conditions are described in the individual experiments. For apoptosis assays, IncuCyte Caspase 3/7 green reagent (Essen BioScience, catalog no: 4440) was added to a final dilution of 1:1,000. Phase contrast images of the cells in standard culture conditions were obtained at 3- to 6-hour intervals using an IncuCyte S3 or IncuCyte Zoom (Essen BioScience). Green fluorescence images were additionally captured for apoptosis assays. Experiments were repeated minimally in technical and biologic triplicates.

### Statistical Analyses

PRISM software was used to plot individual data points and the SD. Single comparisons were performed through the use of an unpaired, two-tailed Student *t* test. For the analysis of differences between multiple groups, ANOVA analysis with Tukey multiple comparisons test was utilized.

### RNA-Seq

The University of Pittsburgh Health Sciences Sequencing Core at the UPMC Children's Hospital of Pittsburgh measured RNA quantity and quality using Qbit (Thermo Fisher Scientific), and performed mRNA library preparation and RNA-seq using an Illumina platform. Fastq files were prepared for RNA-seq analysis using the programs FastQC version 0.11.5 and Fastp version 0.20.0, and salmon version 0.13.1 was used for quantification of transcript expression. Transcript-level estimates were imported into R, version 4.03, using package tximport, version1.18.0. Differential gene expression analysis was performed with package DESeq2, version 1.30.0. Gene-set enrichment analysis was performed using package fgsea, version 1.16.0, which was also used for figure generation along with package EnhancedVolcano, version 1.8.0.

### Data Availability Statement

The RNA-seq data generated in this study are publicly available in Gene Expression Omnibus (GEO) at GSE 182677. In addition, publicly available data generated by others were used by the authors as follows:

#### Cancer Cell Line Encyclopedia Analysis

Ewing sarcoma cell lines were queried in the Broad Institute Cancer Cell Line Encyclopedia (CCLE) database (https://portals.broadinstitute.org/ccle). The somatic mutations in each cell line were compared against genes involved in DNA damage repair and classified as pathogenic, likely pathogenic, variant of unknown significance, benign, or likely benign using COSMIC.

#### PEDS MiONCOseq Data Analysis

PEDS-MiONCOseq is an institutional review board (IRB)-approved, pediatric precision oncology pediatric cohort enrolling since May 2011 as previously described (17). Targeted exome-sequencing data from this cohort of 747 pediatric oncology patients were queried for germline variants in *BARD1.* Pathogenicity status of variants identified was annotated by searching the ClinVar database (https://www.ncbi.nlm.nih.gov/clinvar/).

#### St. Jude Cloud PeCan Data Analysis

The St. Jude Cloud PeCan (Pediatric Cancer Knowledgebase; https://www.stjude.cloud, doi:https://doi.org/10.1101/2020.08.24.264614) germline sequencing data from 1,120 patients (18) were queried for germline variants in *BARD1* and corresponding pathogenicity status of variants was annotated by searching the ClinVar database (https://www.ncbi.nlm.nih.gov/clinvar/).

## Results

### The Landscape of Germline *BARD1* Variants in Pediatric Oncology

Germline variants in DNA damage repair genes are reported in >10% of patients with Ewing sarcoma (9, 10). Clinically, such germline variants are often first discovered when tumors are sequenced upon relapse, as patients with Ewing sarcoma currently do not routinely undergo germline testing at diagnosis. We have previously reported a patient with Ewing sarcoma who harbors a heterozygous pathogenic germline *BARD1* variant (c.176_177AG; p.E59Afs*8) that results in a frameshift that introduces a premature stop codon within the RING domain of BARD1 (ref. 11; Table 1, footnote “^a^”, index case). The RING domain is the BARD1 interaction site for BRCA1. The premature stop codon eliminates the Ankyrin repeat domain and BRCT (BRCA1 C Terminus) domain, the binding site for PAR (19).

**TABLE 1:** The landscape of germline BARD1 variants in a cohort of pediatric oncology patients.

Cancer type	Location	Effect	Sequence change	Amino acid change	VAF	LOH
Leukemia/Lymphoma						
B-cell ALL	chr2:215661812	Missense	c.188T>C	p.L63S	44%	NO
T-cell ALL	chr2:215645930	Missense	c.668A>G	p.E223G	47%	NO
B-cell ALL	chr2:215674215	Missense	c.79G>C	p.E27Q	44%	NO
B-cell ALL	chr2:215674267	**Deletion**		p.R5_N9del	63%	NO
T-cell ALL	chr2:215645322	Missense	c.1276C>G	p.H426D	59%	N/A
Brain Tumors						
Ependymoma	chr2:215645939	Missense	c.659T>C	p.L220S	47%	UPD
Ependymoma	chr2:215657104	Missense	c.281A>C	p.D94A	51%	YES
DIPG	chr2:215645939	Missense	c.659T>C	p.L220S	45%	N/A
Low-grade glioma	chr2:215595215	**Frameshift**	c.1921C>T	p.R641fs	25%	NO
Neuroblastoma						
Neuroblastoma	chr2:215595169	Missense	c.1967G>A	p.G656D	42%	NO
Neuroblastoma	chr2:215632365	Missense	c.1409A>G	p.N470S	51%	NO
Neuroblastoma	chr2:215595181	**Frameshift**	c.1935_1954dup	p.E652fs	31%	NO
Neuroblastoma	chr2:215645966	**Stopgain**	c.632T>A	p.L211X	46%	NO
Bone sarcoma^a^						
Osteosarcoma	chr2:215645471	Missense	c.1127C>T	p.S376L	49%	NO
Ewing sarcoma	chr2:215645877	Missense	c.721T>C	p.S241P	55%	NO
Osteosarcoma	chr2:215645351	Missense	c.1247T>G	p.L416R	32%	NO
Other						
Retinoblastoma	chr2:215645514	Missense	c.1084T>G	p.C362G	42%	N/A

NOTE: Germline sequencing data from PEDS MiONCOseq and St. Jude PeCan datasets was queried for *BARD1* variants and the pathogenicity status of the individual variants were determined. Variants highlighted in blue are of unknown significance. Variants highlighted in red and bolded are pathogenic or likely pathogenic.

Abbreviations: ALL, acute lymphoblastic leukemia; LOH, loss of heterozygosity; N/A, data not available; UPD, uniparental disomy; VAF, variant allele frequency.

^a^Index case: patient with Ewing sarcoma and a germline pathogenic variant in *BARD1:* c.176_177AG; p.E59Afs*8

Pathogenic germline *BARD1* variants provide a moderate risk for heritable breast cancer and have also been reported in pediatric patients diagnosed with high-risk neuroblastoma (20–22). A study of 4,469 patients with breast cancer in Germany revealed 23 (0.51%) with BARD1 loss-of-function (LOF) variants as compared with 0.1% with LOF variants in controls (22). To better understand the frequency of germline *BARD1* mutations across pediatric malignancies, we investigated the landscape of germline *BARD1* variants via analysis of two pediatric oncology sequencing databases: PEDS-MiONCOseq (747 patients at the time of analysis) and St. Jude Cloud PeCan (1,120 patients; ref. 18; Table 1). Four pathogenic or likely pathogenic germline *BARD1* variants (those which are capable or likely capable of disrupting function/causing disease, denoted in red) were found in patients diagnosed with pediatric cancers including neuroblastoma, glioma, and B-cell acute lymphoblastic leukemia. Our index case of the first report of a patient with Ewing sarcoma and a germline pathogenic variant in *BARD1* is listed in the footnote (a) for reference. Fifteen patients with pediatric cancer with variants of uncertain significance (those for which insufficient or conflicting data exists to determine whether they are disease causing, VUS, denoted in blue) were identified, including three patients diagnosed with bone sarcomas. In summary, out of 1,867 patients assessed from two institutions, 15 patients (0.8%) were identified with a *BARD1* VUS and 4 patients (0.21%) with a pathogenic/likely pathogenic germline *BARD1* variant. Twenty-four benign or likely benign germline *BARD1* variants (those unlikely or not the cause of dysfunction/disease) were also noted in this pediatric cancer patient cohort and are included in Supplementary Table S1 for completeness.

### PSaRC318: A Patient-Derived Ewing Sarcoma Cell Line Harboring a Germline Frameshift Variant in *BARD1*

**FIGURE 1:**
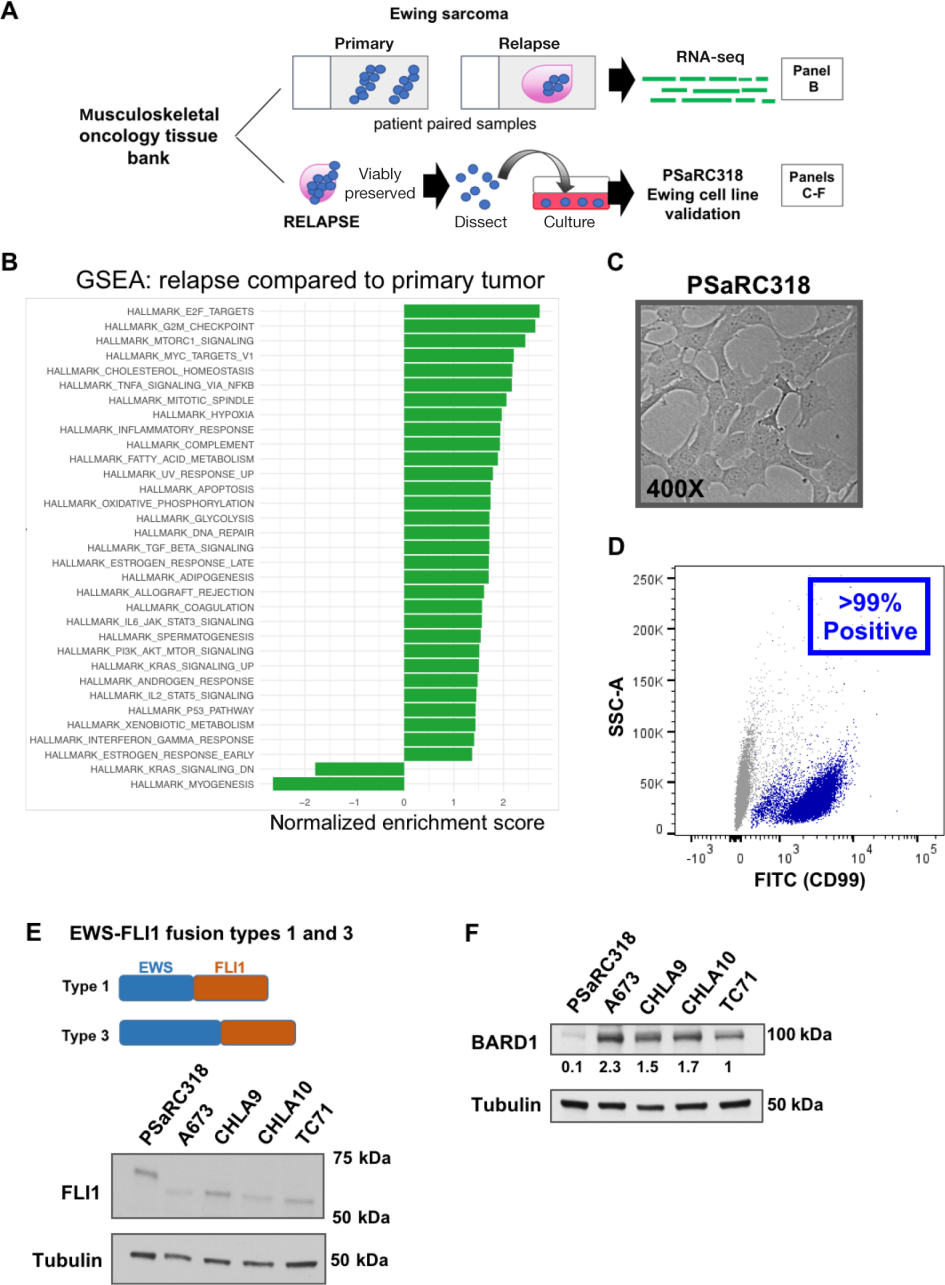
GSEA analysis and validation of a primary cell line from a Ewing tumor with a BARD1 pathogenic variant. **A,** Schematic overview of tumor samples associated with analyses in **B**–**F**. **B,** Gene-set enrichment analysis (GSEA) of RNA-seq data comparing the lung relapse of the Ewing tumor with a germline BARD1 pathogenic variant to the original primary/pretreatment biopsy. Genesets significantly impacted (*P* < 0.05) are included. **C,** Phase contrast image (400×) of the PSaRC318 Ewing tumor cell line. **D,** Flow cytometry showing presence of surface CD99 expression in the PSaRC318 cell line. **E,** Schematic detailing the difference between Type 1 and Type 3 EWS-FLI fusions (top) and Western blot analysis with anti-FLI1 antibody of Ewing sarcoma cell lines with type 3 (PSaRC318) versus type 1 (A673, CHLA9, CHLA10, and TC71) EWS-FL1 fusions. **F,** Western blot demonstrating BARD1 protein expression in the same Ewing sarcoma cell lines as in **E**. PSaRC318 cells demonstrate significantly (*P* < 0.05) less BARD1 expression as compared with other Ewing cell lines. Densitometry values below the blot indicate relative expression values. Experiments in **D**–**F** were completed minimally in biological triplicate.

The EWS-FLI1 fusion oncoprotein itself contributes to impaired DNA damage repair, broadly resulting in the inclusion of Ewing sarcoma as one of the “BRCAness” tumors (3, 4). EWS-FLI1 can interact with BARD1 (23). It is unknown whether the presence of a germline pathogenic variant in a DNA damage repair gene such as *BARD1* is able to further disrupt DNA damage repair and enhance sensitivity of Ewing tumor cells to DNA-damaging agents. Thus, to address this question, we established and validated a patient-derived Ewing sarcoma cell line (PSaRC318) harboring a germline frameshift pathogenic variant in *BARD1* (11). Our workflow of analyzing the relapsed bulk tumor and generating PSaRC318 is summarized in Fig. 1A. RNA-seq analysis was performed on FFPE tumor samples from both the primary and relapsed tumor. Gene-set enrichment analysis (GSEA) was preformed to determine pathways significantly (*P* < 0.05) up- or downregulated in the relapse as compared with the original primary, pretreatment biopsy specimen (Fig. 1B). In addition to DNA damage–related signatures, we noted a number of hallmark immunoregulatory pathways upregulated upon relapse including, but not limited to, the inflammatory and IFNγ response signatures.

Next, viably frozen tumor tissue from a biopsy of the relapsed lung tumor specimen was used to develop a novel Ewing sarcoma cell line, PSaRC318, the morphology of which is shown in Fig. 1C. Flow cytometry confirmed that more than 99% of PSaRC318 cells express surface CD99, a commonly used marker to identify Ewing sarcoma cells (Fig. 1D). Sequencing from the tumor upon relapse revealed a rare Type 3 EWS-FLI1 fusion. Type 3 fusions contain a larger N-terminal portion of EWS (exon 10 breakpoint) as compared with the more common Type 1 EWS-FLI1 fusion (exon 7 breakpoint; see Fig. 1E, schematic). Expression of the Type 3 fusion oncoprotein (with higher molecular weight) was confirmed via anti-FLI1 Western blot analysis of PSaRC318 protein lysate as compared with Ewing cell lines harboring a type 1 EWS-FLI1 fusion (Fig. 1E). In comparison with other Ewing sarcoma cell lines (A673, TC71, CHLA9, and CHLA10), PSaRC318 cells demonstrate significantly less BARD1 expression upon densitometry analysis (*P* < 0.05; Fig. 1F).

### Loss of BARD1 Enhances Ewing Tumor Cell Sensitivity to DNA Damage

**FIGURE 2:**
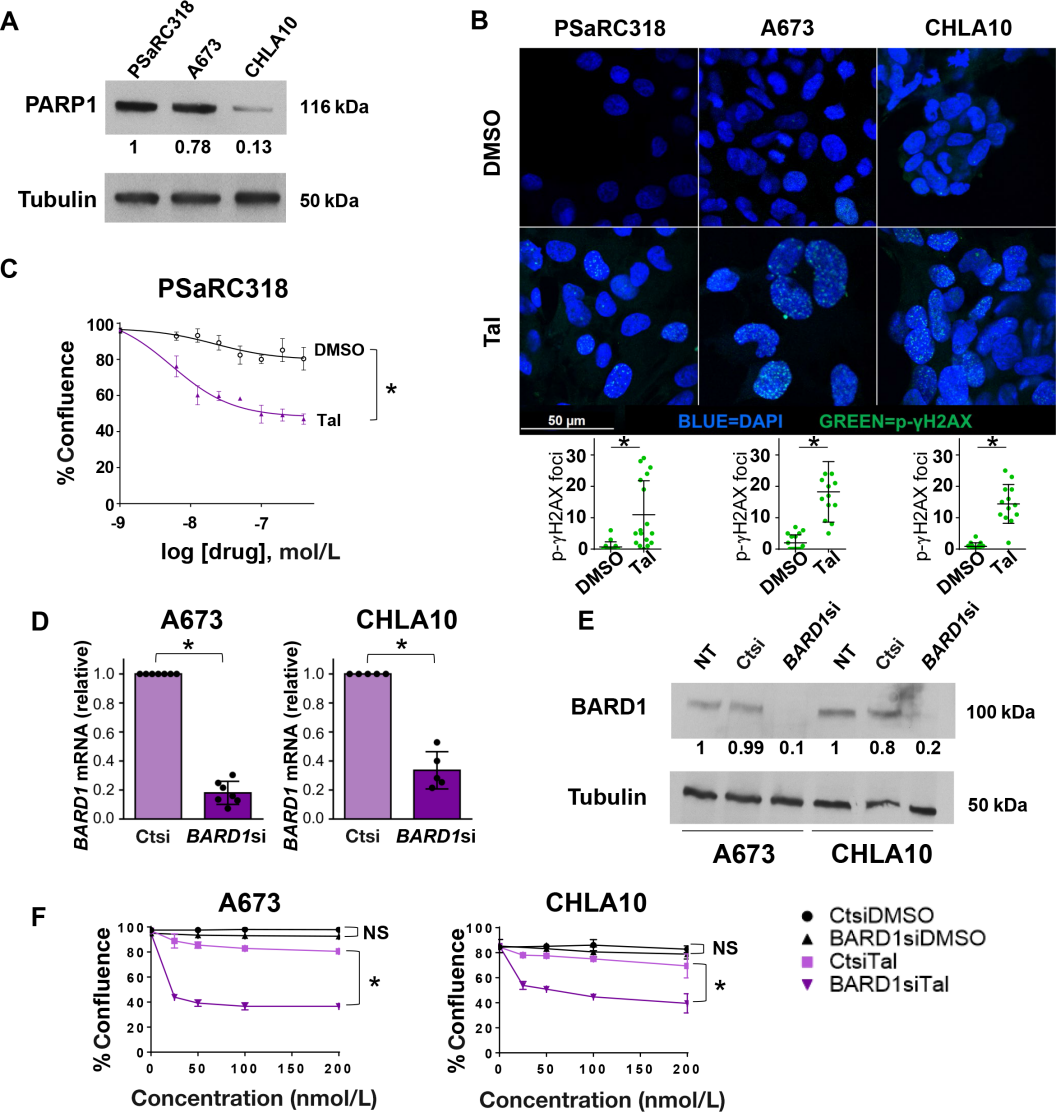
Loss of BARD1 enhances Ewing sarcoma cell sensitivity to PARP inhibition. **A,** Western blot analysis for PARP1 expression in Ewing sarcoma cells. **B,** p-γH2AX and DAPI immunofluorescence staining of PSaRC318, A673, and CHLA10 cells treated with DMSO or 100 nmol/L talazoparib (Tal). Cells imaged at 630× and p-γH2AX foci were quantified (bottom graphs). **C,** IncuCyte assay comparing the confluence of PSaRC318 cells treated with DMSO versus 100 nmol/L talazoparib over 1 week. **D,** qRT-PCR showing *BARD1* mRNA expression in A673 or CHLA10 cells treated with control (ctsi) or BARD1 (*BARD1*si) siRNA. **E,** Western blot analysis for BARD1 expression in untreated (NT) A673 or CHLA10 cells or cells treated with Ctsi or *BARD1*si. **F,** IncuCyte monitoring of cell confluence at increasing concentrations of talazoparib versus DMSO controls in A673 and CHLA10 cells treated with Ctsi versus *BARD1*si. A673 and CHLA10 cell data is graphed at the 60-hour time point. Normalized expression values from densitometry analyses are included under Western blots. Experiments were completed minimally in biological triplicate. NS, not significant; *, *P* < 0.01. Error bars, SD.

Using the validated PSaRC318 Ewing sarcoma cell line as a tool, we next sought to address the impact of BARD1 loss on Ewing cell sensitivity to DNA damage. Prior *in vitro* studies have shown that Ewing sarcoma cells demonstrate sensitivity to PARP inhibition (6, 7), although resistance mechanisms do exist (24). PARP inhibitors act by preventing the recruitment of protein complexes key in DNA damage repair to sites of single-strand breaks (25). Given the pathogenic, frameshift variant present in *BARD1* in the PSaRC318 cells, we questioned whether PSaRC318 cells would demonstrate sensitivity to PARPi. We confirmed PARP1 expression in PSaRC318, A673, and CHLA10 Ewing sarcoma cells (Fig. 2A). Next, we sought to verify that PARPi treatment–induced DNA damage in our model system. PSaRC318 cells were treated with 100 nmol/L talazoparib (vs. DMSO control) for 24 hours, fixed, and then labeled for phospho-γH2AX (p-γH2AX), a marker of DNA double-strand breaks. Talazoparib treatment did indeed induce p-γH2AX staining (punctate dots)/DNA double-strand breaks in PSaRC318 cells as compared with cells treated with DMSO alone (Fig. 2B). p-γH2AX expression was also determined by Western blot analysis (Supplementary Fig. S1A). To determine the impact of talazoparib treatment on the growth and survival of PSaRC318 cells, live-cell IncuCyte analysis was performed to monitor PSaRC318 cell confluence following treatment with increasing doses of talazoparib versus equivalent volume DMSO (vehicle control). We found that PSaRC318 cell proliferation was significantly (*P* < 0.01) impaired by talazoparib treatment (Fig. 2C).

Next, to more directly determine the impact of BARD1 loss on Ewing cell sensitivity to PARP inhibition, we evaluated the effect of siRNA-mediated knockdown of BARD1 in A673 and CHLA10 Ewing cells. CHLA10 cells, a Ewing sarcoma cell line derived post-chemotherapy/upon disease progression, are known to be relatively less sensitive to PARPi as compared with other Ewing cells. Of note, many pathogenic germline variants in DNA damage repair genes are heterozygous, with some degree of loss of heterozygosity demonstrated within tumors (26, 27). Thus, we specifically chose a pooled *BARD1* siRNA-based approach to mimic this scenario by significantly reducing but not eliminating *BARD1* expression (see Supplementary Fig. S1B for BARD1 knockdown efficiency using the SMART pool as compared to the four individual siRNAs). Efficient (70%–80%) knockdown of BARD1 was achieved in both A673 and CHLA10 cells as demonstrated by qRT-PCR and Western blot analysis (Fig. 2D and E; *P* < 0.01). In addition, induction of DNA damage upon talazoparib treatment of A673 and CHLA10 cells was confirmed via p-γH2AX staining (Fig. 2B; Supplementary Fig. S1A for Western blot analysis). Treatment of Ewing cells with talazoparib does not result in loss of BARD1 expression (Supplementary Fig. S1C). Live-cell monitoring of A673 and CHLA10 cells treated with BARD1siRNA revealed enhanced sensitivity to talazoparib as compared with cells treated with control siRNA (Ctsi; Fig. 2F, *P* < 0.01). Together, these results strongly suggest that loss of BARD1 enhances Ewing cell sensitivity to PARP inhibition.

**FIGURE 3:**
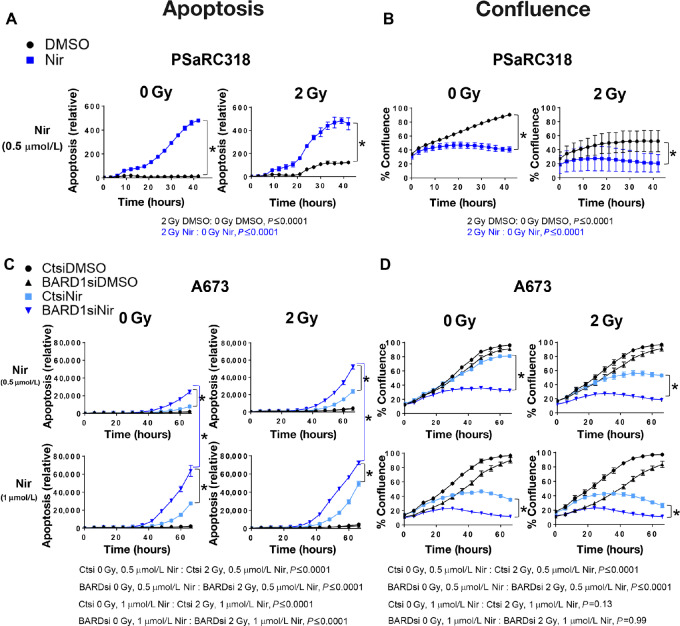
BARD1 loss enhances Ewing sarcoma cell apoptosis in response to niraparib plus radiation. **A,** Relative apoptosis (caspase 3/7 activity) data from IncuCyte assays showing the effect of 0.5 μmol/L niraparib (Nir) versus DMSO control plus either 0 or 2 Gy radiation on PSaRC318 cells. **B,** Confluence data from IncuCyte assays showing the effect of 0.5 μmol/L niraparib versus DMSO control plus either 0 or 2 Gy radiation on PSaRC318 cells. **C** and **D,** A673 cells were treated with control (Ctsi) or BARD1 (*BARD1*si) siRNA, niraparib (at doses indicated) versus DMSO control, and either 0 or 2 Gy radiation and monitored via IncuCyte apoptosis assay (**C**) or confluence assay (**D**). For these experiments, cells were seeded in the presence of niraparib and radiation was performed at 12–15 hours. Relative apoptosis (caspase 3/7 dye activity) is calculated as green fluorescence in μm^2^ divided by confluence. Experiments were completed minimally in technical and biological triplicates. *, *P* < 0.01 as determined by ANOVA analysis with Tukey multiple comparisons test. Error bars, SD.

The addition of DNA damage in the setting of PARPi has been shown to enhance apoptosis of Ewing cells (28). We next wanted to determine the impact of reducing BARD1 expression on Ewing cell response to a combination of direct DNA damage (radiation) plus PARPi. We performed this analysis using a second PARPi (niraparib; ref. 29), which is also being tested in clinical trials for patients with Ewing sarcoma (SARC025, NCT02044120). PSaRC318 cells were treated with 0.5 μmol/L niraparib or DMSO control and then treated with or without 2 Gy radiation at 15 hours and monitored via IncuCyte. PSaRC318 cells demonstrate significantly more apoptosis and are less confluence over time (hour 20, 25, 30, etc.) in the setting of niraparib plus radiation as compared with niraparib alone (*P* < 0.0001; Fig. 3A and B). Of note given the clinical use of radiation in the treatment of Ewing sarcoma, PSaRC318 cells also demonstrate sensitivity to 2 Gy radiation alone (*P* < 0.0001).

To more precisely examine the role of BARD1, we compared the response of A673 and CHLA10 cells treated with Ctsi versus BARD1siRNA to niraparib/radiation. A673 cells were treated with 0.5 or 1 μmol/L niraparib and CHLA10 cells were treated with 1 or 1.5 μmol/L niraparib and then monitored over time via IncuCyte analysis. The higher dose of niraparib was used for CHLA10 cells given their relative resistance to PARPi. At approximately 12 hours, cells were treated with 2 Gy radiation. In A673 cells, cells treated with BARDsi demonstrated more apoptosis as compared with Ctsi-treated cells. In addition, significantly more apoptosis was noted over time (hour 40, 50, 60) when comparing BARDsi cells treated with 2 Gy as compared with 0 Gy at both doses of niraparib tested (*P* ≤ 0.0001, Fig. 3C). BARDsi cells treated with niraparib at either 0.5 or 1 μmol/L doses fail to achieve 50% confluency by 60 hours (Fig. 3D). Multiple statistical comparisons between cell treatment groups are included below the graphs in Fig. 3A–D for reference. Similar results were seen with CHLA10 cells (Supplementary Fig. S2), although as noted, higher doses of niraparib were required to achieve this effect. Together, these data demonstrate that reduction of BARD1 in Ewing sarcoma: (i) enhances sensitivity to both talazoparib and niraparib, (ii) leads to increased apoptosis in the setting of PARPi plus direct DNA damage (radiation).

### Enhanced GBP1 Expression is Noted in the BARD1-Deficient Ewing Cell RNA Landscape

**FIGURE 4:**
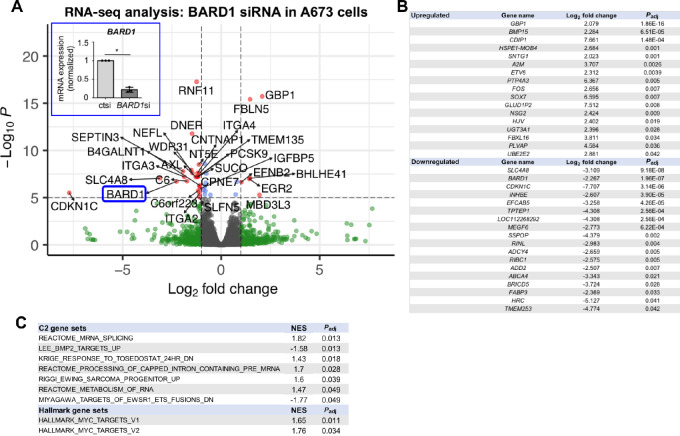
Impact of BARD1 loss on the Ewing sarcoma cell transcriptome. **A,** Volcano plot of genes up-/downregulated upon loss of *BARD1* as compared with A673 Ewing sarcoma cells transfected with Ctsi in three biological replicates. Horizontal dashed lines denote *P* = 0.05 (5 on −log_10_ scale). Vertical dashed lines denote a value of 1 on the log_2_ scale. *BARD1* is circled in blue and highlighted as to verify that it is significantly downregulated upon RNA-seq analysis. In addition, the inset image demonstrates RT-PCR analysis of *BARD1* expression as a second means by which to validate reduction of *BARD1* expression in these RNA samples (×3 biological replicates) as compared with Ctsi-treated cells, *, *P* < 0.05; error bars, SD. **B,** List of most significantly (*P*_adj_) upregulated and downregulated genes with a log_2_ fold change of 2 or greater when comparing cells treated with *BARD1* siRNA versus Ctsi. **C,** Pathway analysis (C2 and Hallmark genesets) of *BARD1* siRNA-treated cells as compared with Ctsi-treated cells. NES, normalized enrichment score; *P*_adj_, adjusted *P* value.

Having determined that Ewing sarcoma cells deficient in BARD1 demonstrate enhanced susceptibility to DNA damage, we next wanted to better understand the changes that occurred in the Ewing sarcoma cell transcriptome upon reducing *BARD1* expression. To do this, we utilized an unbiased approach by performing RNA-seq analysis on RNA isolated from A673 Ewing sarcoma cells treated with control or *BARD1* siRNA for 72 hours. Knockdown of BARD1 was confirmed via RT-PCR prior to RNA-seq (Fig. 4A, inset, *P* < 0.05). Analysis of the resultant RNA-seq data comparing BARD1 knockdown to control cells was performed, using cutoffs of 1 for log_2_ fold change in RNA expression and a *P* value of <0.05 (5 on the −log_10_*P* scale) to generate the volcano plot shown in Fig. 4A. In total, 162 genes were significantly (*P*_adj_ < 0.05) upregulated and 271 genes were significantly downregulated when comparing *BARD* siRNA-treated cells to controls (Ctsi). Of these significant (*P*_adj_ < 0.05) genes, 17 upregulated genes and 18 downregulated genes demonstrated a log_2_ fold change of 2 or greater (Fig. 4B). Pathway analysis of C2 and Hallmark gene sets was also performed and all significantly (*P*_adj_ < 0.05) impacted gene sets and the corresponding normalized enrichment scores (NES) are included (Fig. 4C). Interestingly, expression of guanylate-binding protein 1 (GBP1), a protein implicated in the regulation of cellular response to inflammation and chemotherapy responsiveness (30), was the most significantly upregulated gene upon knockdown of BARD1 (*P*_adj_ = 1.86E-16). GBP1 expression is associated with enhanced motility of lung cancer cells, treatment resistance in ovarian cancer, and better recurrence-free survival in breast cancer. GBP1 functions as a tumor suppressor in colorectal cancer, and in contrast, can protect prostate carcinoma cells from IFNγ-mediated apoptosis (30–34). Given the upregulation of *GPB1* in the BARD si-treated cells upon RNA-seq analysis, we sought to determine whether GBP1 is expressed in PSaRC318 cells.

### GBP1 is Expressed and Contributes to DNA Damage Response in PSaRC318 Cells

**FIGURE 5:**
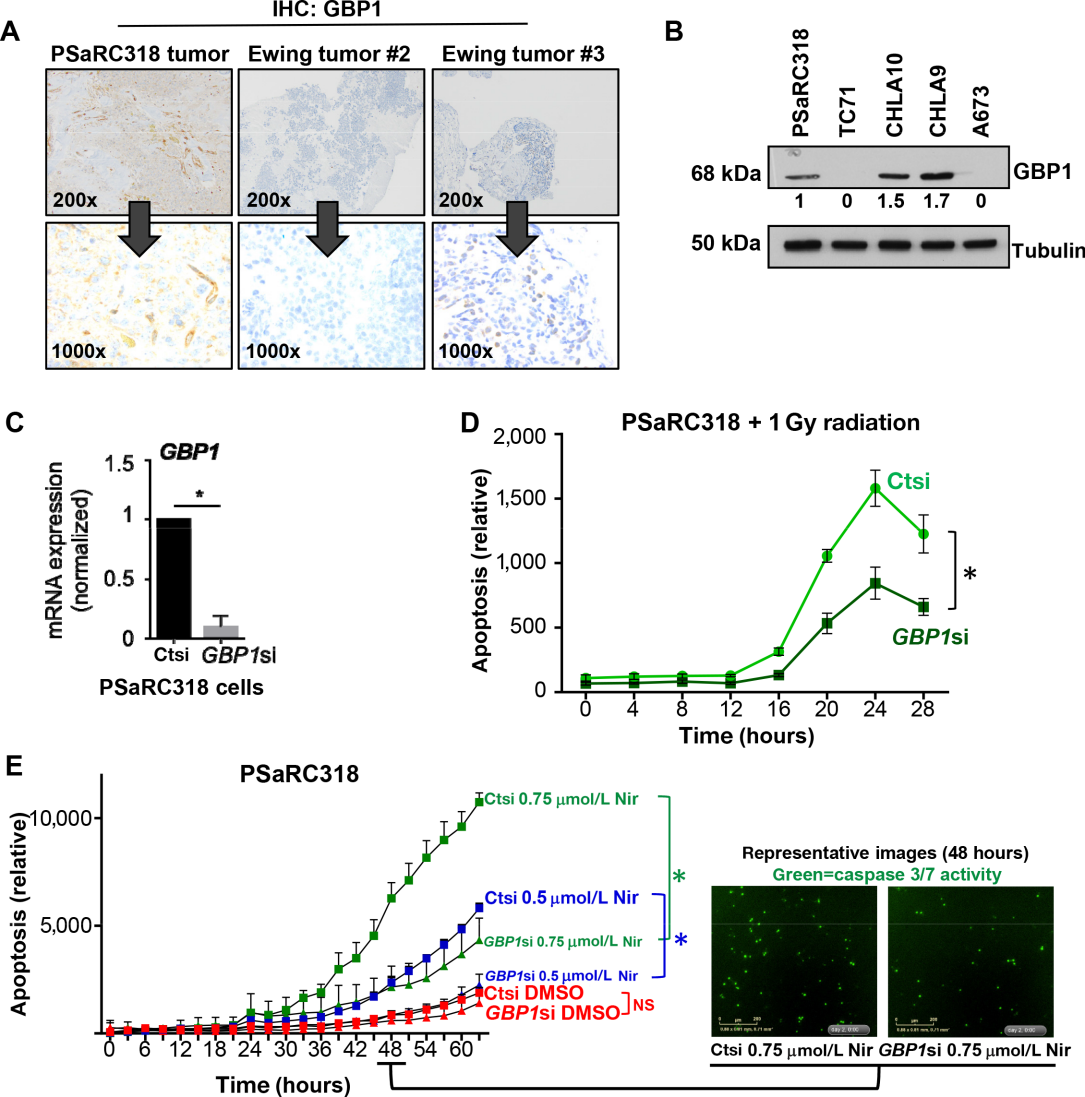
Guanylate-binding protein 1 (GBP1) contributes to Ewing cell sensitivity to DNA damage noted upon loss of BARD1. **A,** IHC analysis of GPB1 expression in the PSaRC318 patient tumor and in two additional independent Ewing tumors. Images provided at both low (200×) and high (1,000×) power. **B,** Western blot for GBP1 in Ewing sarcoma cell lines. Numbers under the blot indicate normalized expression as determined by densitometry analysis. **C,** Normalized *GPB1* mRNA expression of PSaRC318 cells treated with control (ct) or *GBP1* siRNA (si). *, *P* < 0.0001. **D,** PSaRC318 cells were transfected with control or *GBP1* siRNA for 72 hours. Cells were then seeded into 96-well plates in quadruplicate, allowed to adhere and then radiated (dose = 1 Gy). Live-cell IncuCyte monitoring of cell confluence and apoptosis (caspase 3/7 activity) was monitored. The graph displays the relative apoptosis (apoptosis/confluence) in control versus *GBP1* siRNA-treated cells over time. *, *P* < 0.05. **E,** PSaRC318 cells treated with siRNA and seeded as in **D** and then treated with DMSO, 0.5 μmol/L niraparib (Nir), or 0.75 μmol/L niraparib. The graph displays the relative apoptosis (apoptosis/confluence) over time. Representative IncuCyte images at 48 hours are included (right). NS, not significant; *, *P* < 0.05. Experiments completed minimally in biological triplicate. Error bars, SD.

Using IHC to stain for GBP1 expression, a FFPE tissue sample from the original PSaRC318 tumor did indeed demonstrate diffuse cytoplasmic staining for GBP1 (Fig. 5A, left). Additional Ewing tumors were also stained (Fig. 5A, Ewing tumor #2 and #3) for GBP1 and demonstrate no expression (middle) or intercellular heterogeneity in expression (right). To determine whether GBP1 protein is expressed in Ewing sarcoma cell lines, PSaRC318, A673, TC71, CHLA 9, and CHLA10 cell lysates were subjected to Western blot analysis for GBP1. PSaRC318 cells also express GBP1 protein. A673 and TC71 do not express GBP1. CHLA9 and CHLA10 (cell lines from pretreatment and postprogression samples from the same patient) demonstrate GBP1 expression (Fig. 5B). The GBP1 promoter harbors three p53 response elements, an IFN-stimulated response element (ISRE), IFNγ activation site, and a c-Rel site. IFN exposure and NFκB pathway signaling can upregulate GBP1 expression and factors such as VEGF and TGFβ have been reported to downregulate GBP1 expression (30). In cells with no baseline GBP1 protein expression, we demonstrate that expression can be induced upon exposure to IFNγ in both Ctsi- and *BARD1* si-treated cells (Supplementary Fig. S3). We conclude that loss of BARD1 expression is only one of many possible factors contributing to GBP1 expression in Ewing sarcoma.

Given the varied effects of GBP1 in cancer as detailed earlier, we next sought to determine the functional role(s) of GBP1 expression in PSaRC318 cells. To manipulate GBP1 expression, PSaRC318 cells were transfected with Ctsi (control) or *GBP1* siRNA. Figure 5C demonstrates >75% knockdown of *GBP1* mRNA expression (*P* < 0.05). Ctsi- and *GBP1* si-treated PSaRC318 cells were then subjected to radiation and monitored for apoptotic activity in real time (IncuCyte). As shown in Fig. 5D, loss of GBP1 expression results in a significant reduction in relative radiation-mediated apoptosis (apoptosis/cell confluence) of PSaRC318 cells (*P* < 0.05). Nonnormalized confluence data comparing Ctsi and *GBP1*si cells is included in Supplementary Fig. S4. In addition to reduced relative apoptosis in *GBP1* si-treated cells upon radiation treatment, we also noted this effect in GBP1si cells treated with niraparib (Fig. 5E, *P* < 0.05). GBP1 expression in some adult carcinomas alters cell motility. Using scratch/wound healing assays, we noted no difference in cell motility between Ctsi- and *GBP1* si-treated cells (Supplementary Fig. S5). In summary, we find that GBP1 expression is an important factor in the apoptotic response of PSaRC318 cells upon induction of direct DNA damage (radiation) or PARPi.

## Discussion

Relapsed and metastatic Ewing sarcoma remains a deadly disease, and to date, personalized medicine approaches have remained elusive, in part due to the rarity of this cancer. While any single germline DNA damage repair gene variant in patients with Ewing sarcoma is rare, as highlighted by our analysis of *BARD1* germline variants in the PEDS MiONCOseq and St. Jude PeCan datasets, in aggregate, patients with Ewing sarcoma and pathogenic germline variants in DNA damage repair genes constitute approximately 10%–13% of the Ewing sarcoma patient population (9, 10). The prognostic impact of these pathogenic germline variants on patient outcomes is currently unknown. Therapeutically, it is logical to determine whether Ewing tumors harboring pathogenic germline DNA damage repair gene variants demonstrate enhanced sensitivity to agents which induce DNA damage. The Pediatric MATCH Screening Trial experimental subprotocol H (NCT03155620) enrolls patients with pathogenic *ATM*, *BRCA1*, *BRCA2*, *RAD51C*, and *RAD51D* variants to receive the PARP inhibitor olaparib. In this work, we have generated and utilized the novel patient-derived *BARD1*-mutated Ewing sarcoma cell line PSaRC318 as a model to ask the question: can pathogenic variants in the DNA damage repair gene *BARD1* enhance sensitivity to PARPi/DNA damage beyond the vulnerability already imparted by the presence of EWS-FLI1? Indeed, here we show that disrupting BARD1 expression enhances Ewing cell apoptosis in response to PARPi alone and in combination with radiation. On the basis of our current work, we propose the inclusion of patients harboring pathogenic *BARD1* variants in similar future clinical trials aimed at testing DNA-damaging agents/combinations in patients with any one of many pathogenic germline variants in DNA damage repair genes such as *ATM*, *BRCA1*, *RAD51C*, etc. and Ewing sarcoma. Better understanding germline DNA damage defects in patients with Ewing sarcoma and discovering novel approaches to treat Ewing tumors with additional impairments in DNA damage repair is an ongoing priority of our work.

Knowing the presence of additional DNA damage repair gene variants is also important when selecting Ewing cell lines to test new agents/effects. Using Cancer Cell Line Encyclopedia (CCLE, Broad Institute) sequencing data, we determined the presence of somatic variants in DNA damage repair genes in the 14 Ewing sarcoma cell lines used in the original analyses of PARP inhibition in Ewing sarcoma cells (6, 7). Ewing sarcoma cell lines such as SKPNDW and ES7 harbor pathogenic somatic variants in *FANCM* (Fanconi anemia, complementation group M). In addition, the Ewing cell line CADOES1 harbors a pathogenic somatic *SLX4* variant (Supplementary Table S2). Both of these genes are involved in DNA damage repair (35, 36) and cells with such additional dysfunctional DNA damage repair may behave differently than Ewing sarcoma cells without such defects.

Through our RNA-seq studies of BARD1 loss, we uncovered a link to GBP1 expression. As noted in the results, GBP1 expression can be regulated by a host of factors and some Ewing cell lines, such as CHLA10, express GBP1 in the absence of BARD1 deficits. We were able to demonstrate that GBP1 expression contributes to the apoptotic response to DNA damage in Ewing sarcoma. On the basis of these findings, future studies to determine the association of Ewing tumor GBP1 expression/expression patterns with patient outcome are warranted.

In addition to GBP1 upregulation, we noted additional genes in our RNA-seq analysis of *BARD1* knockdown cells that could also contribute to DNA damage/chemotherapy response, such as *CDKN1C.* We noted a significant downregulation of cyclin dependent kinase inhibitor 1C (CDKN1C, also known as P57Kip2) upon knockdown of *BARD1.* CDKN1C expression is strongly induced by EWS-FLI1 (37, 38) and is reported to play a role in chemoresistance. One report has demonstrated that BARD1 can physically interact with EWS-FLI1 (23), and thus it is interesting to speculate how the loss of BARD1, or loss of other BARD1-binding partners such as BRCA1, may impact the transcriptional activity of EWS-FLI1. Our findings could indicate that loss of BARD1 leads to a reduction of EWS-FLI1–induced *CDKN1C* expression.

Finally, given the significant cross-talk between DNA damage and immunoregulatory pathways, novel DNA-damaging agent/immunotherapy combinations are also worthy of preclinical testing specifically in this unique subset of Ewing tumors. GSEA analysis of our RNA-seq data from the PSaRC318 tumor at relapse demonstrated upregulation of multiple immunoregulatory pathways including TNFα/NFκB, inflammatory response, TGFβ, IL6, IL2, and IFNγ (Fig. 1). Ongoing studies in our laboratory are assessing how concomitantly targeting DNA damage repair and immunoregulatory pathways may be an effective alternative approach for treating Ewing tumors harboring additional germline DNA damage repair defects.

## Supplementary Material

Supplemental Table S1 Benign germline BARD1 variants.

Supplemental Figure S1 Additional protein/mRNA expression data (corresponding to Figure 2).

Supplemental Table S2 Prevalence of somatic DNA damage repair gene variants in Ewing cell lines included in original studies of PARP inhibition.

Supplemental Figure S2 BARD1 loss enhances Ewing sarcoma cell apoptosis in response to niraparib plus radiation in CHLA10 Ewing sarcoma cells.

Supplemental Figure S3 Interferon-gamma-induced GBP1 expression in A673 Ewing sarcoma cells.

Supplemental Figure S4 PSaRC318 cell confluency data.

Supplemental Figure S5 Loss of GBP1 expression does not impact PSaRC318 cell migration.
