# Supplementary material for: BRCA1-Associated RING Domain-1 (BARD1) Loss and GBP1 Expression Enhance Sensitivity to DNA Damage in Ewing Sarcoma
Source: Cancer Res Commun. 2022 Apr 20;2(4):220–32. doi: 10.1158/2767-9764.CRC-21-0047 (PMC9524505; doi:10.1158/2767-9764.CRC-21-0047)
Supplement: Supplemental Table S1 — Benign germline BARD1 variants. [file crc-21-0047-s01.pdf]

Supplemental Table S1

Cancer Type	Location	Effect	Sequence Change	Amino Acid Change
LEUKEMIA/LYMPHOMA				
B-cell Lymphoma	chr2:215593522	Missense	c.2212A>G	p.I738V
Acute Lymphoblastic Leukemia	chr2:215595164	Missense	c.1972C>T	p.R658C
Acute Lymphoblastic Leukemia	chr2:215593522	Missense	c.2212A>G	p.I738V
Acute Lymphoblastic Leukemia	chr2:215595164	Missense	c.1972C>T	p.R658C
Acute Myeloid Leukemia	chr2:215595164	Missense	c.1972C>T	p.R658C
Acute Myeloid Lukemia	chr2:215595164	Missense	c.1972C>T	p.R658C
Leukemia	chr2:215593522	Missense	c.2212A>G	p.I738V
Burkitt lymphoma	chr2:215610518	Missense	c.1738G>A	p.E580K
BRAIN TUMORS				
Astrocytoma	chr2:215674261	Missense	c.33G>T	p.Q11H
Atypical Teratoid/Rhabdoid Tumor	chr2:215595164	Missense	c.1972C>T	p.R658C
Glioma, Brain Stem	chr2:215610518	Missense	c.1738G>A	p.E580K
Glioblastoma	chr2:215593522	Missense	c.2212A>G	p.I738V
Glioma, Brain Stem	chr2:215595164	Missense	c.1972C>T	p.R658C
DIPG	chr2:215595164	Missense	c.1972C>T	p.R658C
Choroid Plexus Carcinoma	chr2:215593522	Missense	c.2212A>G	p.I738V
NEUROBLASTOMA				
Neuroblastoma	chr2:215674261	Missense	c.33G>T	p.Q11H
Neuroblastoma	chr2:215674261	Missense	c.33G>T	p.Q11H
BONE SARCOMA				
Rhabdomyosarcoma	chr2:215595164	Missense	c.1972C>T	p.R658C
Osteosarcoma	chr2:215593522	Missense	c.2212A>G	p.I738V
Osteosarcoma	chr2:215595164	Missense	c.1972C>T	p.R658C
OTHER				
Angiosarcoma	chr2:215593522	Missense	c.2212A>G	p.I738V
Wilms tumor	chr2:215595164	Missense	c.1972C>T	p.R658C
Wilms tumor	chr2:215593543	Missense	c.2191C>G	p.R731G
Squamous Cell carcinoma	chr2:215593522	Missense	c.2212A>G	p.I738V

benign/likely benign

Supplemental Table S1.

Benign germline *BARD1* variants. Benign or likely benign germline *BARD1* variants identified in the PEDS MiONCOseq and St. Jude PeCan datasets. This serves as an extension of the data presented in **Table 1**.
