# Supplementary material for: BRCA1-Associated RING Domain-1 (BARD1) Loss and GBP1 Expression Enhance Sensitivity to DNA Damage in Ewing Sarcoma
Source: Cancer Res Commun. 2022 Apr 20;2(4):220–32. doi: 10.1158/2767-9764.CRC-21-0047 (PMC9524505; doi:10.1158/2767-9764.CRC-21-0047)
Supplement: Supplemental Figure S1 — Additional protein/mRNA expression data (corresponding to Figure 2). [file crc-21-0047-s02.pdf]

# Supplemental Figure S1

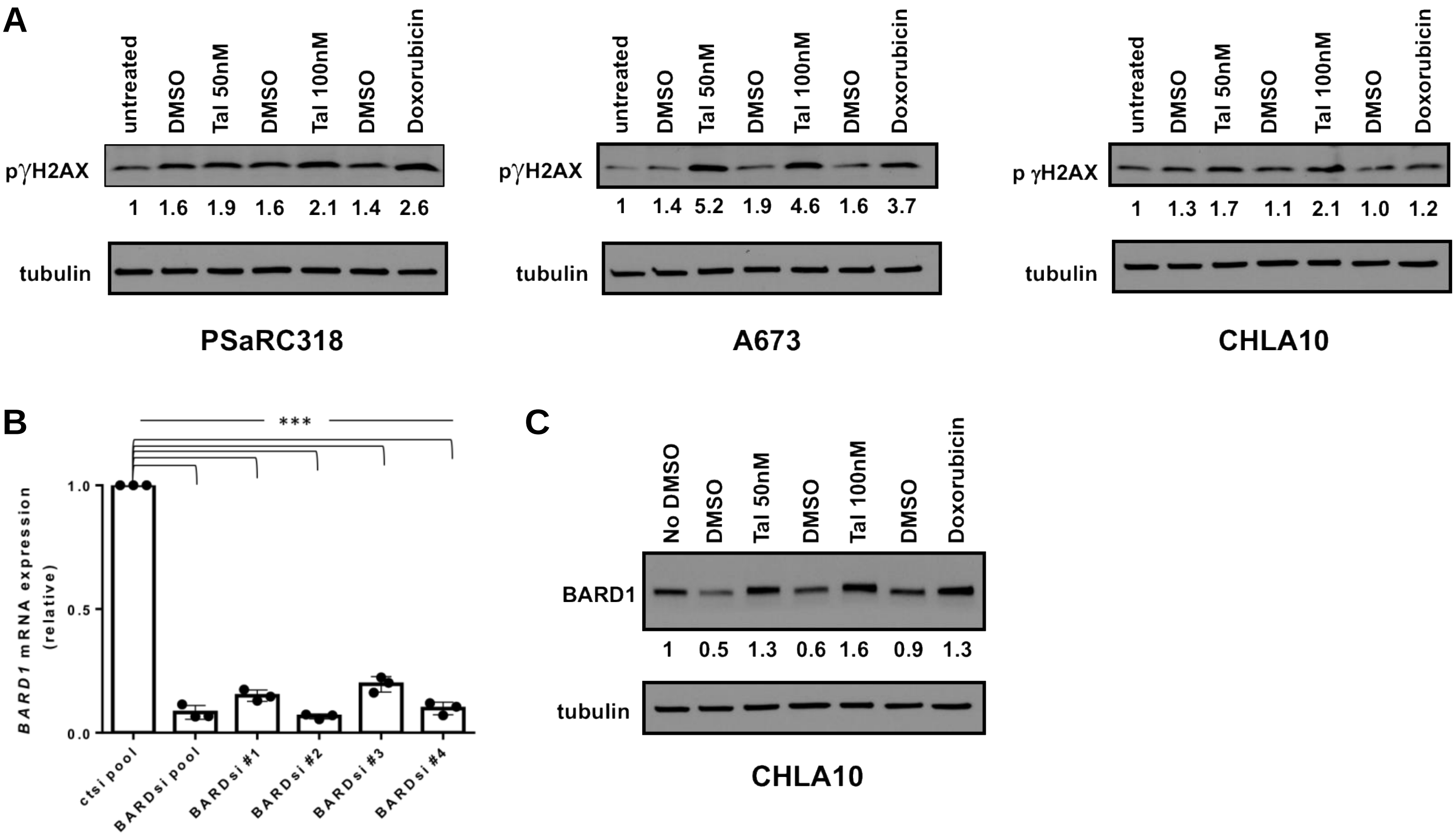


**Supplemental Figure S1**.

**Additional protein/mRNA expression data (corresponding to Figure 2). A,** Western blots for pγH2AX in PSaRC318, A673, and CHLA10 Ewing sarcoma cells under the treatment conditions noted. These data correspond to the immunofluorescence data for pγH2AX in Figure 2B. **B,** A673 Ewing sarcoma cells were transfected with SMART pool control (non-targeting) siRNA (ctsi), SMART pool BARDsiRNA, or one of four individual BARD1siRNAs from the pool (#1-#4) for 72 hours. Resulting RNA(cDNA) was subjected to RT-PCR analysis for *BARD1*. **C,** Western blot for BARD1 expression in CHLA10 cells treated with DMSO, Talazoparib (Tal), doxorubicin (20nM) or untreated. Values under western blots in **A** and **C** represent relative expression as determined by densitometry analysis. Experiments were performed minimally in biological triplicate. *** denotes $p < 0.0001$, error bars denote S.D.
