# Supplementary material for: BRCA1-Associated RING Domain-1 (BARD1) Loss and GBP1 Expression Enhance Sensitivity to DNA Damage in Ewing Sarcoma
Source: Cancer Res Commun. 2022 Apr 20;2(4):220–32. doi: 10.1158/2767-9764.CRC-21-0047 (PMC9524505; doi:10.1158/2767-9764.CRC-21-0047)
Supplement: Supplemental Table S2 — Prevalence of somatic DNA damage repair gene variants in Ewing cell lines included in original studies of PARP inhibition. [file crc-21-0047-s03.pdf]

Supplemental Table S2

cell line	variant	predicted impact	olaparib	talazoparib	niraparib
			sensitivity (rank)		
A673	*TP53*<br>*ERCC2*	pathogenic<br>benign/likely benign	34	31	32
CADOES1	*SLX4*<br>*APC*	pathogenic<br>likely benign	42	13	
SKPNDW	*FANCM*<br>*TP53*	pathogenic<br>likely pathogenic			48
RDES	*TP53*	pathogenic			
MHES1	*TP53*	likely pathogenic	41		22
ES1	*TP53*	pathogenic	23	23	
ES3	*TP53*	likely pathogenic			
ES5	*PTCH2*<br>*TP53*	likely benign<br>pathogenic			
ES6	*TP53*	likely pathogenic			
ES7	*POLE*<br>*FK8P3*<br>*FANCM*<br>*ERCC4*<br>*TP53*	likely benign<br>VUS<br>pathogenic<br>benign/likely benign<br>likely pathogenic	21	18	6
ES8	*TP53*	pathogenic	10	5	8
EW16	*TP53*	likely pathogenic			
EW22	*TP53*	pathogenic	14	8	10
EW24	*APC*<br>*TP53*	benign<br>likely pathogenic			

benign/likely benign
variant of unknown significance
pathogenic/likely pathogenic

Supplemental Table S2.
Prevalence of somatic DNA damage repair gene variants in Ewing cell lines included in original studies of PARP inhibition. Cancer cell line encyclopedia (CCLE) data was queried for somatic mutations in DNA damage repair gene mutations in the 14 Ewing sarcoma cell lines listed. The predicted/known impacts of the somatic mutations and sensitivity rank (sensitivity to a compound as compared to all other cells lines in the CCLE) to the PARP inhibitors olaparib, talazoparib and niraparib are also noted.
