# Supplementary material for: BRCA1-Associated RING Domain-1 (BARD1) Loss and GBP1 Expression Enhance Sensitivity to DNA Damage in Ewing Sarcoma
Source: Cancer Res Commun. 2022 Apr 20;2(4):220–32. doi: 10.1158/2767-9764.CRC-21-0047 (PMC9524505; doi:10.1158/2767-9764.CRC-21-0047)
Supplement: Supplemental Figure S2 — BARD1 loss enhances Ewing sarcoma cell apoptosis in response to niraparib plus radiation in CHLA10 Ewing sarcoma cells. [file crc-21-0047-s04.pdf]

Supplemental Figure S2

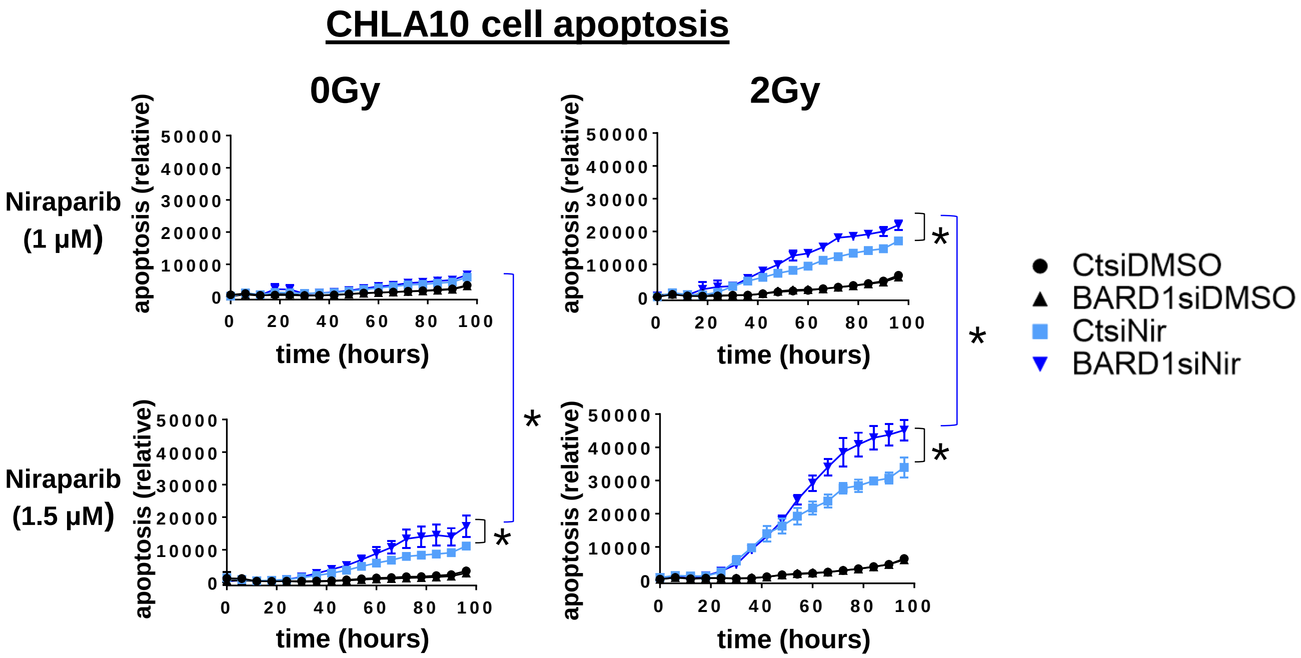

Supplemental Figure S2.
BARD1 loss enhances Ewing sarcoma cell apoptosis in response to niraparib plus radiation in CHLA10 Ewing sarcoma cells. CHLA10 cells were treated with control (Ctsi) or BARD1 (BARD1si) siRNA, Nir (at doses indicated) versus DMSO control, and either 0 or 2 Gy radiation and monitored via IncuCyte apoptosis assay (Caspase 3/7 activity). For these experiments, cells were seeded in the presence of Nir and radiation was performed at 12-15 hours. Relative apoptosis (caspase 3/7 activity) is calculated as green florescence in μM^2 divided by percent confluence. Experiments were completed minimally in biological triplicate. These data correspond with Figure 3 in the main text. * denotes p-value <0.01 as determined by ANOVA analysis with Tukey's multiple comparisons test. Black brackets indicate comparison between CtsiNir and BARDsiNir at the same Nira dose. Blue brackets indicate comparison between BARDsiNir samples at 1 versus 1.5µM doses of Niraparib. Error bars denote S.D.
