# Supplementary material for: BRCA1-Associated RING Domain-1 (BARD1) Loss and GBP1 Expression Enhance Sensitivity to DNA Damage in Ewing Sarcoma
Source: Cancer Res Commun. 2022 Apr 20;2(4):220–32. doi: 10.1158/2767-9764.CRC-21-0047 (PMC9524505; doi:10.1158/2767-9764.CRC-21-0047)
Supplement: Supplemental Figure S3 — Interferon-gamma-induced GBP1 expression in A673 Ewing sarcoma cells. [file crc-21-0047-s05.pdf]

Supplemental Figure S3

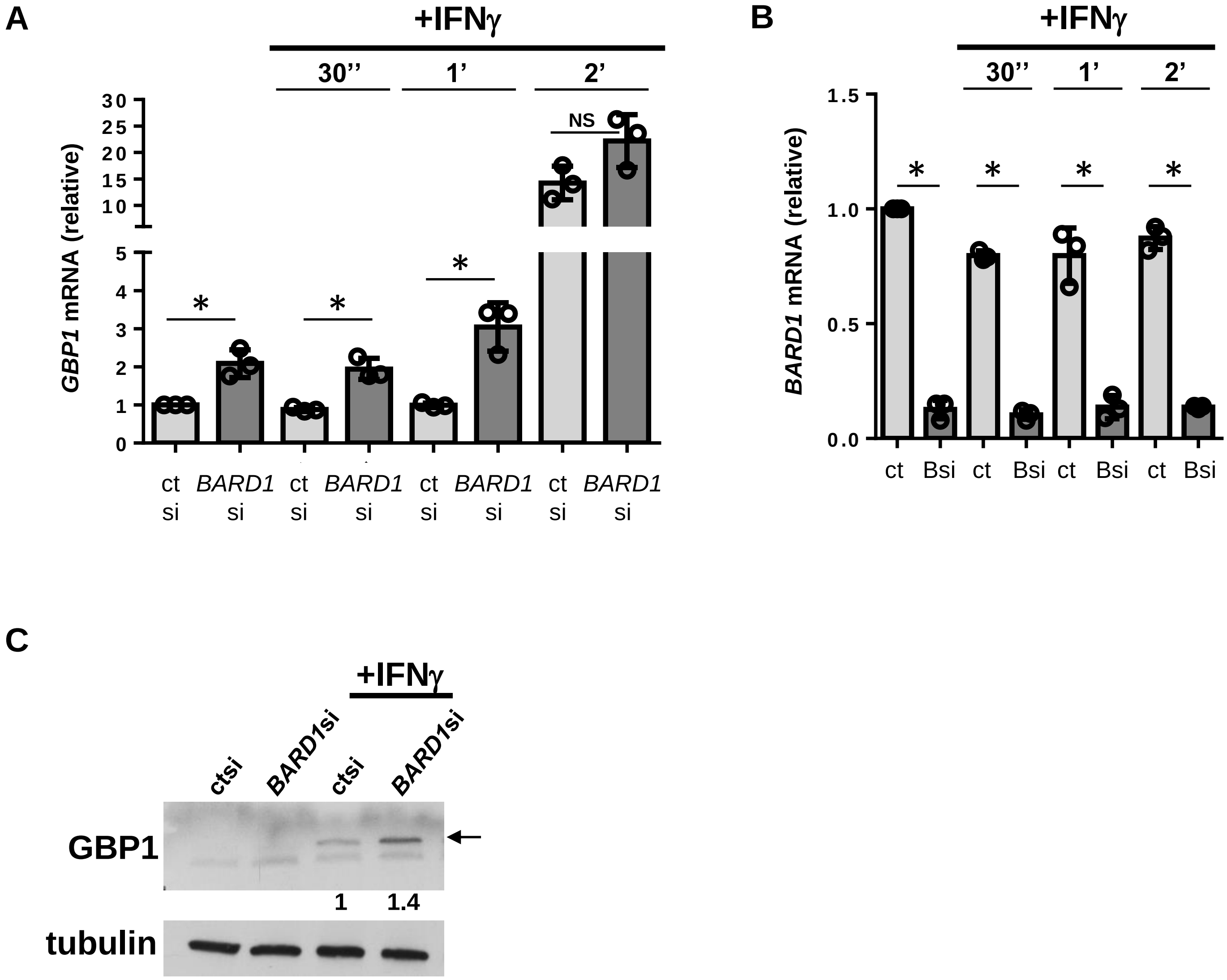

Supplemental Figure S3.

Interferon-γ-induced GBP1 expression in A673 Ewing sarcoma cells. A673 cells were treated with control (ctsi) or *BARD1 (Bsi)* siRNA for 72 hours. Cells were then stimulated with vehicle or 100U/mL IFN-γ for the times indicated and RNA was isolated. qRT-PCR analysis for **A,** *GBP1* and **B,** *BARD1* was performed. **C,** Western blot analysis of GBP1 expression in A673 cells treated with ctsi or BARD1si and vehicle or interferon (+ IFN-γ). Tubulin is included as a loading control. Normalized densitometry values are listed below the blot. Experiments were performed in biological triplicate. * denotes $p<0.05$, NS=not significant ($p>0.05$). Error bars denote S.D.
