# Supplementary material for: BRCA1-Associated RING Domain-1 (BARD1) Loss and GBP1 Expression Enhance Sensitivity to DNA Damage in Ewing Sarcoma
Source: Cancer Res Commun. 2022 Apr 20;2(4):220–32. doi: 10.1158/2767-9764.CRC-21-0047 (PMC9524505; doi:10.1158/2767-9764.CRC-21-0047)
Supplement: Supplemental Figure S4 — PSaRC318 cell confluency data. [file crc-21-0047-s06.pdf]

Supplemental Figure S4

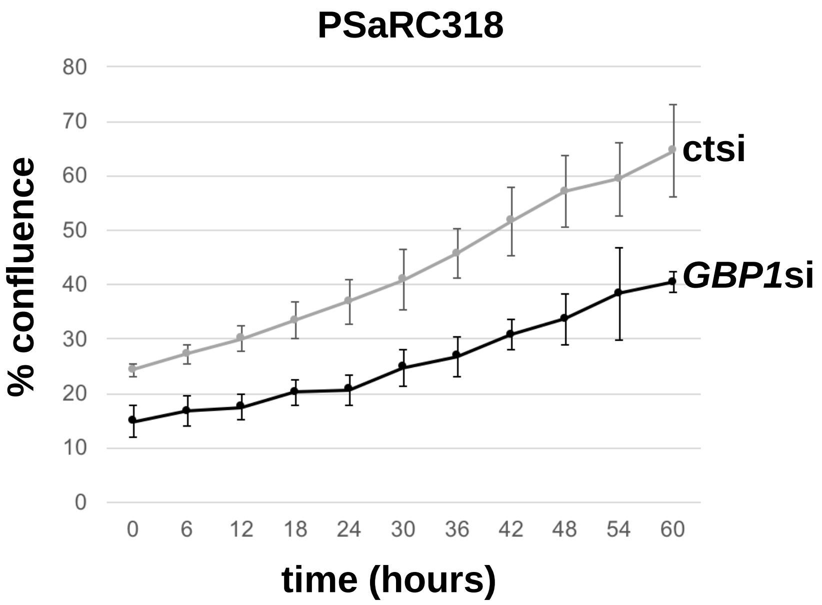

Supplemental Figure S4.
PSaRC318 cell confluency data. PSaRC318 cells were treated with control (ct) or *GPB1* siRNA and then subjected to IncuCyte live cell monitoring over time (data corresponding to Figure 5). Baseline differences in non-normalized cell confluency between ctsi and *GBP1*si-treated PSaRC318 cells is plotted over time. Experiments completed minimally in biological triplicate. Error bars denote S.D.
