# Supplementary material for: BRCA1-Associated RING Domain-1 (BARD1) Loss and GBP1 Expression Enhance Sensitivity to DNA Damage in Ewing Sarcoma
Source: Cancer Res Commun. 2022 Apr 20;2(4):220–32. doi: 10.1158/2767-9764.CRC-21-0047 (PMC9524505; doi:10.1158/2767-9764.CRC-21-0047)
Supplement: Supplemental Figure S5 — Loss of GBP1 expression does not impact PSaRC318 cell migration. [file crc-21-0047-s07.pdf]

Supplemental Figure S5

A

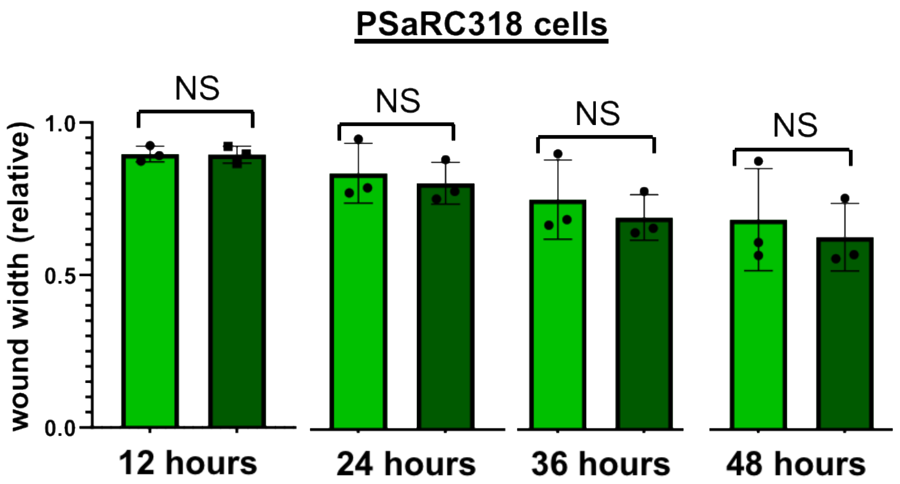

B

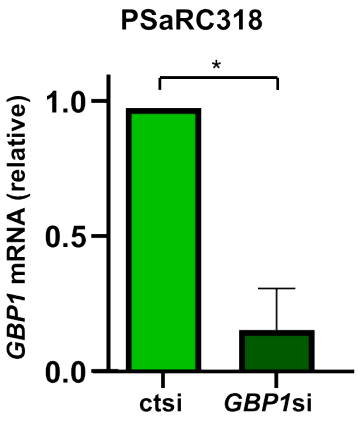

Supplemental Figure S5. **Loss of GBP1 expression does not impact PSaRC318 cell migration.**
A, PSaRC318 Ewing sarcoma cells were transfected with control (ct, light green) or *GBP1* (dark green) siRNA for 48 hours and then seeded into wells of a 96-well plate in technical triplicate and allowed to form a confluent monolayer overnight. A linear scratch was made with the Incucyte 96-well WoundMaker Tool and live-cell monitoring of wound width was performed over time. This graph displays the wound width as a percentage of the initial wound width (width at time=0 hours). **B,** qRT-PCR showing *GBP1* mRNA expression in PSaRC318 cells treated with ctsi or *GBP1* siRNA. Experiments were completed in biological triplicate. NS= not significant, denoting a p-value >0.05. * denotes a p-value <0.05. Error bars denote S.D.
